# Introducing dynamic consent for improved trust and privacy in research involving human biological material and associated data in South Africa

**DOI:** 10.3389/fgene.2024.1272924

**Published:** 2024-04-03

**Authors:** Larisse Prinsen

**Affiliations:** Department of Public Law, University of the Free State, Bloemfontein, South Africa

**Keywords:** biomedical research, dynamic consent, participant-centric initiative, protection of personal information, privacy, trust

## Abstract

Biomedical research using human biological material and data is essential for improving human health, but it requires the active participation of many human volunteers in addition to the distribution of data. As a result, it has raised numerous vexing questions related to trust, privacy and consent. Trust is essential in biomedical research as it relates directly to the willingness of participants to continue participating in research. Privacy and the protection of personal information also influence trust. Informed consent has proven to be insufficient as it cannot overcome the informational deficit between primary and unknown future uses of material and data and is therefore not fully informed and invalid. Broad consent is also problematic as it takes full control of samples and data flow from the research participant and inherently requires that a participant must trust that the researcher will use their material or data in a manner that they would find acceptable. This paper attempts to offer some insight into how these related issues can be overcome. It introduces dynamic consent as a consent model in research involving human biological material and its associated data. Dynamic consent is explained, as well as its claims of superiority in instances where future research is possible. It is also shown how dynamic consent contributes to better control of the samples and data by the research participant, and how trust may be improved by using this consent model. Dynamic consent’s co-existence with and support of the South African Protection of Personal Information Act of 2013 is also assessed. The limitations of dynamic consent are also discussed.

## 1 Introduction

Biomedical research that makes use of human biological material and data is vital for increasing our understanding of biological and molecular mechanisms underlying illness and disease, testing the efficacy of new medications, medical devices and interventions, and for moving towards models of personalised medicine (Budin-Ljøsne et al., 2017).

Biomedical research promises significant societal benefits. However, in order to deliver on this promise, the research community requires the active participation of many human volunteers in addition to the distribution of data. Also, biomedical research requires the continued collection of human biological material samples, health and outcome data from these samples, and also follow ups (Budin-Ljøsne et al., 2017).

Research participants are therefore essential partners in research endeavours since, through their voluntary participation, researchers have access to biological samples and data. These samples and data are vital to research and are available only by generous donation from participants (Horn et al., 2011). These participants may choose to participate in research for varied reasons including receiving the benefits of investigational medication, improving future healthcare practices or contributing to scientific knowledge. However, trust is always a key component of their participation (Horn et al., 2011) and a loss of participant or public trust can threaten continued research that uses human biological material and data (Williams et al., 2015). There are great concerns about the potential abuses and misuses of the collected data (Erlich et al., 2014), such as the invasion of privacy which may involve deeply personal issues. New forms of data and participant-led research are also challenging the traditional mechanisms of oversight and are raising questions about the ethics of partnership and collaboration between research participants and researchers (Tauginienė et al., 2021).

As mentioned above, in order to fulfil the promise of biomedical research, participant involvement is needed as well as analysis of large datasets containing the information of these participants. Sharing this information, however, requires protecting participants from potential harm (Erlich et al., 2014), such as exploitation and confidentiality or privacy breaches. Traditionally, regulatory frameworks have protected the rights and welfare of research participants as passive subjects, relying strongly on paternalistic views that research participants may not be able to assess correctly the risks and benefits involved in the research process or study (Tauginienė et al., 2021). However, because of strong human and consumer rights movements, the protection of human research participants has shifted and is now guided by informed consent or by Institutional Review Board or Research Ethics Committee procedures (Tauginienė et al., 2021). That said, the wide scope and nature of biomedical research that uses human biological material and data challenges a one-size-fits-all approach to obtaining consent. Furthermore, review mechanisms and both informed and broad consent have been shown to be insufficient consent models in biomedical research involving human participants (Prinsen, 2023).

Contemporary data protection models rely mainly on de-identification and de-identified data is largely allowed to flow freely. However, the flow of personal information is restricted and usually explicit consent from the participant allowing the dissemination of information or proof that the risk of re-identification has been minimised, is required (Erlich et al., 2014). De-identification and standard data security measures fall short in three important aspects (Erlich et al., 2014):

1. Standard data security controls may adequately protect data from unauthorised access but may be insufficient against abuses by a legitimate recipient of the information.
2. Advances in re-identification attacks have reduced the utility of de-identification techniques.
3. De-identification does not allow an individual control over data, which is a core element of privacy.

Considering these shortcomings and the limitations of de-identification, participants may, at best, be faced with cumbersome and poorly understood informed consent procedures that attempt to predict the future or, alternatively, ethically problematic broader consent processes (Prinsen, 2023). At worst, they may be given empty promises of anonymity. Researchers and the guardians of data, on the other hand, are then faced with manoeuvring between data utility and privacy (Erlich et al., 2014). This Sisyphean trap may, however, be overcome if trust and trust-enabling frameworks between participants and researchers are established. Such frameworks can be established by following the principles that transparency creates trust, that increased control enhances trust, and that reciprocity maintains trust (Erlich et al., 2014). These principles are discussed in more detail below.

Current data-management discussions frame the value of data against the risks to participants as a zero-sum, meaning that whatever is gained by one side is lost by the other. Erlich et al. (2014) have, however, suggested that a trust-based framework would be advantageous as both research participants and researchers can benefit from data sharing. Dynamic consent, which is discussed below, may be able to support a trust-based framework.

Given the exponential speed at which innovations in technology develop, researchers need flexibility in conducting their research in order to react quickly; thus, traditional approaches to the planning and conducting of biomedical research are unsatisfactory (Budin-Ljøsne et al., 2017). Dynamic consent is a strategy to involve participants, support the principle of informed consent and address the stationary aspect of consent by technological constructs such as communication platforms that establish a continuous two-way communication between researchers and participants (Tauginienė et al., 2021). It is seen as consent which is supported by the necessary information for participants to actively consent to their participation and also consent that is dynamic, regularly revisited, and not static or negotiated in a one-off process (Tauginienė et al., 2021). Dynamic consent may enhance participants’ understanding of research and increase their scientific literacy and thereby positively affect their willingness to remain in a research project (Budin-Ljøsne et al., 2017).

This article therefore discusses trust, dynamic consent and privacy in order to introduce dynamic consent as a mechanism to benefit and improve trust and privacy in biomedical research involving human biological material and associated data.

## 2 Trust

### 2.1 What is trust?

Trust has been described as a mechanism that enables people to deal with situations of risk or uncertainty (Van der Geest et al., 2005). Various forms of trust have been identified, such as general trust and social trust (Van der Geest et al., 2005). However, in the context of biomedical research, two forms of trust are relevant and they depend on the person on whom trust is declared and on the circumstances. The first form of trust is known as “personal trust” and this is trust between two individuals. The second form of trust is trust directed at entities such as institutions, professional bodies or governments, and is referred to as “institutional trust” or “impersonal trust” (Kerasidou, 2017). Regardless of the different forms trust may take, trust relationships share the common characteristics of an assumption of vulnerability by the trustor, an attitude of goodwill by the trustee, and voluntariness (Kerasidou, 2017).

In the context of biomedical research, the trust relationship may be personal (between the research participant and the researcher) or institutional and thus between the research participant and the research institution or between two or more institutions (Kerasidou, 2017).

A further concept in need of clarification is “trustworthiness”. This relates to the person being trusted, the trustee, and the exhibition of characteristics which indicate that this person has goodwill towards the trustor. Trustworthiness is shown by the data controller and trust is given by the participant (Schuler Scott et al., 2019). For people to have trust, institutions must show trustworthiness (Schuler Scott et al., 2019). A person may be seen as trustworthy when they acknowledge the value of the trust vested in them and use that to rationally decide how to act (Kerasidou, 2017). Dynamic consent may be a mechanism of showing goodwill towards a research participant, since researchers are able to openly communicate with participants and show their intentions of acting in the greater good by achieving medical advances, for example.

### 2.2 Why do we need trust in biomedical research?

Some have argued that trust is not necessary in biomedical research since there are numerous instruments, laws, rules and authorities to regulate and oversee all research activities (Kerasidou, 2017). However, studies over the last few decades have found that trust plays an important role in the willingness of persons to participate in health research (Resnik, 2021) and a lack of trust may be seen as a great threat which can jeopardise consenting to participate in biomedical research (Kerasidou, 2017). As mentioned above, human participation in biomedical research is vital and thus trust cannot be regulated away.

Research participation always entails some level of risk to the participant, be it physical or informational, which means that in research involving human participants the participants make themselves vulnerable. Vulnerability and belief in the trustee’s goodwill are the basis of the participant-researcher relationship (Kerasidou, 2017). By becoming a participant, a person surrenders their health and health-related information to the researchers and institutions. Systems of control and regulation do not fully compensate a person for putting themselves in a vulnerable position. This is where trust becomes relevant in that the participants have to trust that the researcher has an attitude of goodwill towards them. In research, this goodwill means that the researcher acknowledges the vulnerability of the participant and takes it into account when considering how to design, conduct and implement their research (Kerasidou, 2017).

Viewing trust as the cornerstone of the participant-researcher relationship suggests a trust-based relationship. As mentioned above, the data utility *versus* privacy challenge may be overcome by establishing a trust-based framework. The first principle in doing so holds that transparency creates trust. This means that transparency between the parties involved is key and that research participants must be informed of not only the intended but also the actual use of data (Erlich et al., 2014). This is a common feature of information privacy statutes and may be seen in the provisions of the Protection of Personal Information Act of 2013, which is discussed in more detail below. It further demonstrates that trust and privacy are interconnected and cannot be separated. The second principle of a trust-based framework holds that increased control enhances trust. Keeping in mind the uncertainties involved in biomedical research, it is virtually impossible to make fully informed decisions about future data uses and risks, but this issue may be overcome when a research participant is given control over the future use of data.

While clear communication about possible risks is critical in ensuring informed consent, current informed consent practices require participants to make one-off decisions about future data sharing with unknown risks. Broad or blanket consent practices are also problematic as the participant has to surrender their control to another person and trust that they will act with goodwill towards the participant. These consent practices further do not accommodate changing privacy preferences over time (Erlich et al., 2014). Dynamic consent is, however, able to do so—as will be explained below. The third principle of a trust-based framework holds that reciprocity maintains trust. This means that mechanisms whereby participants reward researchers who act appropriately by continuing to participate, while punishing those who violate their trust by withdrawing their participation, may provide valuable incentives for win-win behaviour (Erlich et al., 2014) which enables ongoing research. Building on these principles, a participant-centric bilateral consent framework is suggested and it is further suggested that dynamic consent offers such a framework.

A bilateral consent framework, at its core, enables participants to have dynamic control over access to their data. In current consent frameworks the participant delegates control to the researcher who, upon completion of a study, may delegate further use decisions to an Internal Review Board or to Research Ethics Committees. In a bilateral consent framework, the data control remains primarily with the participant. The researcher may then approach the participant with information about secondary uses of the data and the participant may choose to (re-)consent or to revoke and withdraw consent (Erlich et al., 2014). A bilateral consent framework thus engages the participant by making it possible for the researcher to solicit participant data, while at the same time empowering the participant to change their preferences. This framework therefore emphasises reciprocity and agency, envisions data sharing, and sees consent as a shared process which requires iteration and feedback (Erlich et al., 2014). Dynamic consent is a bilateral consent framework.

Trust is important and is influenced by the sense of control and by privacy concerns (Van der Geest et al., 2005). Creating trust gives participants control, and requesting consent is an essential condition for solving privacy issues (Van der Geest et al., 2005).

### 2.3 Consent and trust

The history of biomedical research is marred by scandals such as the experiments conducted by Nazi doctors, the Tuskegee Syphilis Study and the Hwang Woo-suk scandal—to name a few. These incidents have undermined public trust in biomedical research and in an attempt to restore this trust, regulatory instruments, laws and rules have been created, such as The Nuremberg Code of 1948, the 1964 Declaration of Helsinki or the Belmont Report of 1979. In addition, Institutional Review Boards and Research Ethics Committees were created to increase accountability and transparency (Kerasidou, 2017) and consent became a recognised and indispensable requirement for conducting any medical or scientific research involving human participants.

The role of informed consent is to allow participants to make decisions and to safeguard trust in research endeavours (Dankar et al., 2020). Valid informed consent for medical research participation has traditionally required mental capacity to make a reasonable decision, voluntariness and the absence of any form of coercion or undue influence, the provision of information necessary to make a decision, understanding of the given information, and the expression of the decision. When these elements are seen as a whole, it suggests that a consenting person is an autonomous, rational agent making an informed and voluntary decision in line with their own values (Resnik, 2021).

Research has, however, shown that decisions are often not autonomous as the full provision of information has not occurred, the consenting person does not understand the information or other factors may be present which interfere with comprehension (Resnik, 2021). Trust may be seen as a crucial element which compensates for the lack of understanding as it may assure the consenting person that they are not being manipulated, exploited or deceived (Resnik, 2021).

The relationship between trust and consent is reciprocal in that trust may develop during the consent process as the parties become acquainted with one another and form a relationship, but a degree of trust must be presupposed before the consent process begins. Research participants are more likely to participate in research if they have trust in the researchers, the research organisation and the research project itself (Resnik, 2021). It has also been argued that consent and the requirement therefore is a method of trust building (Kerasidou, 2017).

As mentioned above, participants make themselves vulnerable when participating in research. At the start of participation, the participant must give consent and consent lays down the conditions of the relationship between the participant and the researcher. Prospective participants are informed of the risks and possible benefits of the research project, what is required of the participant, and what may be expected from the research, researcher and the research institution. Participants are then given the opportunity to voluntarily make a decision about their willingness to participate or not. The provision of information about the risks and benefits does not, however, absolve researchers of their duty to minimise these risks, to ensure fair distribution of the benefits or to protect the welfare of the participants. In order for participants to trust researchers, they need to believe that the researchers will conduct the research with an attitude of goodwill towards them (Kerasidou, 2017). This means that when a participant gives consent to participation, they do not confirm trust, but rather presuppose it, or differently stated, consent consolidates trust.

Human beings are social animals and trust may come naturally to some, but once trust has been broken it can be difficult to restore. Trust is a form of social capital and, therefore, activities which promote honest, open and respectful communication and dialogue may help in building, maintaining and restoring trust in research (Resnik, 2021). This also means that building, maintaining and restoring trust means doing the same in regards to trustworthiness (Kerasidou, 2017).

Trust may be built through numerous mechanisms, such as developing relationships with participants, demonstrating a track record of accountability, or showing concern for the best interests or goodwill of others. Building trust in biomedical research is essential and becomes more challenging the more distant the research becomes from the participant (Horn et al., 2011). This is often the case when taking into account the unknown secondary and future uses of human biological material and data. Although consent processes alone cannot build trust, it may be considered a minimum effort in terms of doing so (Horn et al., 2011). Consent is essential in nurturing trust but it has been described as the fruit of the tree, rather than its roots (Resnik, 2021).

Trust, control and privacy are strongly related and, accordingly, enabling participants to exert control over their own information may increase trust and thus reduce privacy concerns (Van der Geest et al., 2005). Consent is a mechanism that allows participants to exert control. Control and information are considered central to consent and give rise to two effects. First, participants are informed of relevant matters such as risks or harms and benefits which may flow from participation in research. Second, consent enables participants to exert control and retain responsibility over what they feel is sensitive information. Where consent is used as a process involving participants who control their own personal data, a valuable strategy for dealing with trust and privacy concerns may be found (Van der Geest et al., 2005).

Practices such as informed consent and the use of Institutional Review Boards and Research Ethics Committees have been useful in ensuring the protection of future research while fostering public trust in biomedical research (Kerasidou, 2017). Informed consent, however, is insufficient in biomedical research and broad consent, which was developed to overcome informed consent’s shortcomings, is also problematic as it removes control from the participant (Prinsen, 2023). Dynamic consent may therefore offer a valuable solution to the issue of consent in biomedical research.

## 3 Dynamic consent

New ways of conducting research have given rise to new ethical norms, practices and standards. The status of participants and their level of involvement in research has been a particularly prevalent new question, along with concerns about the appropriate format of consent. A clear shift has also taken place towards more participant-centric initiatives which place the participant in a partnership relationship with the researcher in both the decision-making process and the research study (Steinbekk et al., 2013). Research participants want to be involved, valued and engaged in research and many have shown the desire to be more actively involved in medical research and their health. They further desire transparency and openness and do not wish to be surprised about the use of their donated materials and data (Horn et al., 2011). Participants want to trust that researchers are conducting research with their best interests and goodwill in mind. Various participant-centric research initiatives use emerging technologies to engage participants in new ways and these systems or frameworks enable participants to exercise as much or as little control over their material and information as they prefer. Dynamic consent is one such technology-backed, participant-centric initiative (Horn et al., 2011).

The dynamic consent framework is founded on the work of an expert group that studies legal, social, technical and compliance aspects of consent (Prinsen, 2017) and has the potential to radically alter the nature of consent in research (Kaye et al., 2015) as it supports the flow of new knowledge between the laboratory, clinic, researcher and participant (Mason and O’Neil, 2007). Dynamic consent, with its two underlying concepts of allowing the revocation of consent for data use, and engaging in communication about data use (Schuler Scott et al., 2019), was created to address problems with one-off consent, to develop trust and improve participant recruitment and participation, and is founded on the principles of revocation and engagement (Schuler Scott et al., 2019).

In the past, and sometimes still today, consent was or is obtained in paper format which was then filed away when a person decided to participate in research. Dynamic consent uses electronic systems which enable a participant to keep track of their data, including records of donated human biological material and what this material has been used for. It further allows a participant to monitor and update consent choices over time. For example, a participant may wish to allow the use of their material or data in a new research project or may wish to limit the research which may be conducted using such a sample or data. Accordingly, this model of consent allows control over past and currently donated materials in addition to any future material to be donated (Prinsen, 2017).

### 3.1 The reason for dynamic consent

The requirement that consent be obtained by researchers prior to initiating a proposed study is a fundamental principle of research ethics and law. It has also repeatedly been shown to underpin respect for persons and their autonomy. Consent has become a method of recording individual involvement, and for determining the scope of what is included under consented to activities. Therefore, it may be seen as the formalisation of an implicit social contract between the public and researchers (Kaye et al., 2015). New forms of biomedical research, however, challenge the meaning of informed consent and question the current processes of engaging with participants. The uncertain scope of consent is especially controversial and, in an attempt to address this, broad consent has been suggested as an understandably practical solution. However, for various reasons, a broad consent approach is insufficient for meeting the requirements of meaningful informed consent (Prinsen, 2023).

Unlike traditional research, biomedical research does not follow a single experimental procedure in which participants are asked to participate. Rather, it is a request to participate in an ongoing, continuous inquiry with multiple investigations and methods that involve unknown risks, and it is suggested that new research trends demand new models of consent (Kaye et al., 2012). Differently stated, the consent procedure must also be an ongoing one.

Ethically, it is necessary to enable a participant who has given consent under a set of circumstances to review this consent as new research possibilities, using the same material samples or data, emerge. Also, the possibility exists that research participants may benefit clinically from updated information about their data and samples (Kaye et al., 2012). Legally, by providing a more comprehensive form of consent more bases are covered and thus the legal liabilities which could arise because of the absence of consent are reduced, since the scope of consent is more clearly defined.

As biomedical research changes, so too must the role of the research participant change and evolve (Kaye et al., 2015). Furthermore, individual autonomy is not static and involves changing choices, opinions and preferences. Consent is a mechanism whereby participants’ rights may be protected in research and consent decisions must have the ability to change over time since people are prone to changing their minds (Schuler Scott et al., 2019). Research participants are no longer passive human subjects, but are active and interested participants and consent must now be seen as a process of ongoing interaction between a researcher and a participant.

O’Neil (2006) stated that true consent is reliant on access to extendable information, the concept of rescindable consent, and the right to veto certain activities. Respect for a participant and their autonomy therefore means that participants must be given as many choices and as much control over information, their material and data as possible (Kaye et al., 2015).

Theoretically, dynamic consent benefits both the researcher and the participant, since the participant is given information related to their material, there is transparency regarding their information usage and sharing which enhances trust, and there is the option to revoke consent. This promotes the relationship between research participant and researcher. The researcher benefits from dynamic consent as they gain a business edge by setting best practice, and the relationship with the participant is flexible–meaning that newer and more refined usage may be allowed (Prinsen, 2017).

#### 3.1.1 Dynamic consent as a participant-centric initiative

A participant-centric initiative may be understood as meaning “a tool, program and project that empowers a participant to engage in research processes using IT” (Prinsen, 2017). By making use of an IT interface, it provides an ongoing, continuous interactive means of obtaining consent and maintaining communication between participants and researchers (Kaye et al., 2012). The key characteristics of a participant-centric initiative are that it is based on respect, promotes the empowerment of participants, and is focused on participation. The researcher and the participant are central in decision-making and are equal partners in the research process (Kaye et al., 2012). Participant-centric initiatives therefore greatly emphasise autonomy.

Participant-centric initiatives exhibit four functions. First, they serve a “matchmaking” function which enables the recruitment of research participants. Second, they provide a “direct-to-consumer” service by offering participants genetic testing and analyses and give them the opportunity to participate in research projects (Tamir, 2010). Third, “dynamic control” aids ongoing interaction between the researcher and the participant. Fourth, the initiatives have a “citizen science function” which engages participants in facilitating, designing and executing research projects (Kaye et al., 2012).

Using participant-centric initiatives may greatly benefit research governance by ensuring adherence to basic ethical and legal principles, improving recruitment methods, and maximising participant retention. They may also minimise costs, enhance knowledge and understanding of the research process, and encourage and sustain public trust through greater involvement, accountability and transparency. Participant-centric initiatives are able to achieve these benefits by streamlining the consent process, decreasing the need for de-identified data, facilitating participant recruitment, facilitating participant retention, promoting the delivery of better quality healthcare, improving the quality of research, and sustaining public trust and confidence in research since greater involvement in research has a dual effect. It firstly improves knowledge of the research process and secondly ensures transparency and accountability on the part of the researcher. Research may then be conducted at a higher standard and will be in tune with societal expectations and concerns, which will result in enhanced public confidence and trust (Kaye et al., 2012).

### 3.2 The meaning of dynamic consent

As suggested by the name, dynamic consent is dynamic in that given consent is changeable and adaptable. This idea, however, has a narrow and a broad meaning:

• In a narrow sense, it is a personalised communication platform which enables greater participant engagement in research by enabling an interactive relationship between researchers and participants (Steinbekk et al., 2013). Researchers must foster a relationship of confidence, understanding and trust to establish true insight into what is at stake in the course of research. Dynamic consent may be defined as a new approach to engaging persons in the use of their information and material.
• In a broader sense, it is also an interactive and personalised platform which enables participants to engage in research as much or as little as they prefer and to amend their consent decisions in real time (Kaye et al., 2015).

Dynamic consent is seen as dynamic in that it enables the giving and revocation of consent to the use of materials or data, it centralises transactions and interactions, allows participants to be approached for different projects or feedback, and allows for consent processes to be modified over time (Schuler Scott et al., 2019). At its core, dynamic consent is a mechanism of enabling communication between participant and researcher, and offers the participant the opportunity to be continually informed and in control of their information and material (Wee et al., 2013).

Dynamic consent as a participant-centric initiative places research participants at the centre of the decision-making process by providing an interactive IT interface. It is a dynamic approach as it allows interaction over time, enables renewal of consent to new projects, enables consent to be amended in real time as circumstances change, and gives participants the confidence that their amendments will have an effect (Kaye et al., 2015). When a person initially agrees to any processing of their personal material or data, they may do so without fully understanding the implications of what they are consenting to. After some time, they may wish to review or revoke the initial consent in order to create a new agreement which is more in line with their preferences. With dynamic consent, a participant may control the use and flow of their data and material and change their consent about what is permitted and what is not.

Dynamic consent has certain characteristic features. The first is that it comprises different consents. It is not locked in time at the onset of a project and, depending on the nature of the research project, participants are able to consent to a wide range of uses of their material and data, or they may choose to be approached on a case-by-case basis or may create varying preferences for different research types. These preferences may be opt-in or opt-out and the participant is therefore able to tailor his profile to receive certain information at certain times (Kaye et al., 2015).

The second feature of dynamic consent pertains to its tailored aspect. Since a dynamic consent interface acts as a personalised communication forum, a source of information and a platform on which consent may be modified, all aspects of the interface may be tailored to the preference of the participant. Persons may choose how and when they are to be contacted and what information they wish to receive (Williams et al., 2015)—which emphasises an improvement in control exerted by participants.

The third feature of dynamic consent involves the customisation of research needs. This consent model clearly incorporates a flexible design able to accommodate researchers and participants. All aspects of the interface may be tailored to the proposed project and in this manner extend the interaction between the parties (Kaye et al., 2015).

Dynamic consent improves trust in how data is used, as control of the data is passed to the participant (Schuler Scott et al., 2019). From the features discussed above and the repetitive emphasis of tailoring to the preferences of the participant, it should become obvious why and how dynamic consent may improve trust in biomedical research. Where the participant can tailor their experience, they are the controllers thereof–meaning that they are able to trust the experience.

### 3.3 Dynamic Consent’s benefits and claims of superiority

In understanding, recognising and supporting biomedical research as a partnership between researchers and participants, dynamic consent enables research while also improving the research experience. Dynamic consent therefore offers participants engagement in the process, better respects their autonomy, and also offers meaningful consent. Researchers derive benefit from engaged participants, streamlined participant recruitment, and improved trust. Legally, dynamic consent is valuable as it offers better protection by eliminating ambiguity and vagueness. Ethically, dynamic consent may also be seen as beneficial since it allows for the true expression of autonomy (Prinsen, 2017).

In addition to these benefits and improved trust and privacy as discussed in this article, dynamic consent is also beneficial in providing for the facilitation of efficient re-contact, conformity to the highest legal standards, fine-grained withdrawal mechanisms, the enabling of better communication, improved scientific literacy, and transparency and risk management.

#### 3.3.1 Facilitation of efficient re-contact

Re-contact is often impractical. Dynamic consent offers a method of easy re-contact with participants which grants them accessible information and allows the participant to make an informed decision (Kaye et al., 2015). Maintained contact with participants assists researchers in addressing numerous ethical and legal issues which may arise in unforeseen circumstances. Dynamic consent has also been touted as being able to overcome other ethical challenges encountered in biomedical research (Tauginienė et al., 2021). However, a full discussion thereof falls outside the scope of this article.

#### 3.3.2 Conformity to the highest legal standards

Freely given consent is universally regarded as a requirement of biomedical research as seen in legal and regulatory documents across the globe. Dynamic consent provides a flexible and responsive mechanism of addressing changing legal and ethical requirements (Tauginienė et al., 2021). It may even provide better protection of autonomy than current international standards (Kaye et al., 2015). It is in this flexibility that dynamic consent can accommodate the slightest change in circumstances associated with the consent and therefore the fine-grained functioning of dynamic consent is also seen as beneficial. This also benefits trust as legal compliance and sensitivity may lead to participants feeling better protected.

#### 3.3.3 Fine grained withdrawal

Research participants have the right to withdraw their consent, material or data by requesting that it not be made available for certain secondary or future research projects or even that it be destroyed. Dynamic consent enables a more nuanced choice by offering more information and preference-related options to a participant and, in doing so, excludes the zero-sum “all or nothing” mode of withdrawal which is often found in withdrawal circumstances (Kaye et al., 2015). This not only improves retention of participants but also trust.

#### 3.3.4 Enablement of better communication

Traditionally, consent procedures involve an initial engagement session with the participant at the start of the research project but rarely provide for mechanisms of continued communication (Mascalzoni et al., 2009). In addition, research findings are seldom conveyed to the participants. Dynamic consent uses an online personalised consent and communication platform in order to facilitate the consent process and two-way, ongoing communication between researchers and participants (Budin-Ljøsne et al., 2017). Dynamic consent enables the return of findings according to the participant’s selected preferences. It also creates a means whereby broader engagement may be nurtured, which extends beyond an information sheet. This adds value to the research study (Kaye et al., 2015).

#### 3.3.5 Improved scientific literacy

By implementing a user-friendly and accessible platform, dynamic consent gives participants additional opportunities to gain knowledge and understanding of the information provided in their own time. Participants are granted time for reflection and consideration and they are thus empowered to control the type and amount of information they receive and when they wish to receive it. This may lead to a more realistic understanding of research as an interactive and long-term process, may improve participant confidence by transparency and accountability which leads to improved trust, and may support the development of appropriate expectations of what research may achieve (Kaye et al., 2015).

#### 3.3.6 Improved transparency and risk management

Transparency and accountability may be improved by dynamic consent as the research process, the use of material or data and consent may be traced throughout all the studies. This therefore provides for operational control over risk (Kaye et al., 2015). Participants may also be contacted about controversial issues, such as the protection of personal information, and in this manner trust is safeguarded.

In addition to these benefits, dynamic consent presents six claims of its superiority over other forms of consent (Steinbekk et al., 2013):

1. Dynamic consent offers greater respect for participant autonomy than other consent models as it is better able to meet the specifications of autonomy embedded in specific or informed consent requirements. Dynamic consent enables participants to exercise their autonomy by providing consent to new types of research, in real time, as opposed to once-off consent (Kanellopoulou et al., 2011). This means that since participant preferences are used as the point of departure when establishing potential uses of their material and data, participants are given the opportunity to consent to primary and secondary uses of their material and data.
2. Participants are kept better informed by dynamic consent. The ability to keep participants informed is seen as essential in all research consent processes, and dynamic consent is better suited to fulfil the ideals of disseminating detailed information (Whitley et al., 2012). Additional information may appeal to participants who wish to have control or who are uncertain about the specifics of what they are participating in.
3. Dynamic consent also claims to be a solution to other biomedical research-related challenges such as participant recruitment and retention (Budin-Ljøsne et al., 2017). The dynamic consent model encourages participation in biomedical research. Since trust is created by transparency and accountability, proponents of dynamic consent argue that it will positively affect not only participant recruitment but also retention—and this ultimately results in sustainable biomedical research (Kaye et al., 2012). Also, dynamic consent addresses any criticism that participants are regarded as a mere supply of biological material as the participant becomes an active partner in research (Saha and Hurlbut, 2011). Furthermore, public insight and knowledge are increased by dynamic consent. However, it may be argued that possible participants may be deterred by being confronted with, or even intimidated by, all the details and complexities of biomedical research and then being asked for consent over and over again. As a result, dynamic consent may be described as a two-edged sword in the context of participant recruitment. It could increase trust since participants are given different choices and trust is raised by transparency, and the participant’s sense of control is also increased. In addition, it seems that reciprocity is amplified since dynamic consent accommodates the return of information. On the other hand, participants may then have exaggerated hopes and expectations of what a research project could yield. When these hopes are not realised, trust may be breached and recruitment may decrease.
4. Dynamic consent transfers control to the participant. Concerns about the lack of participant control over both the research and the results are addressed (Wagstaff, 2011). This may be the strongest argument in favour of dynamic consent and may potentially lead to new participant rights (Whitley, 2009).
5. Ethical responsibility is transferred from Research Ethics Committees to participants. This would result in a move towards an open and democratic scientific process which ensures socially robust knowledge. Since new consent must be provided for new research projects, the need for Ethics Review Boards is eliminated or decreased (Kaye, 2012).
6. Dynamic consent enables the return of results and incidental findings in an easy, user-friendly and tailored manner. Proponents of dynamic consent argue that the return of results and findings is necessary as it respects participant autonomy as well as reciprocity and beneficence (Steinbekk et al., 2013).

### 3.4 How dynamic consent works

Dynamic consent as a consent platform is achieved by using technical solutions, compliance services and legal accountability (Prinsen, 2017). Dynamic consent therefore entails a new digital system which allows participants to give consent electronically and by offering dynamic consent along with online services participants can monitor the possible uses of their material samples and personal information or data and make decisions about how these may be used in future (Prinsen, 2017).

Dynamic consent works in a reciprocal fashion where the research participant or data subject is approached to participate in a research project and is provided with relevant information about the project by the researcher or data controller. The participant then consents to the project. During the course of and after the completion of a project, the researcher provides feedback on the findings of the project or notifies the participant of new enquiries or uses of their donated materials or data and again provides the relevant information to the participant. The participant is then given the opportunity to change their preferences which may mean re-consenting to the secondary uses, revoking their consent, or withdrawing from the study. Once these changed preferences are received by the researcher, they can adjust their actions accordingly. This process is continual and facilitates keeping the participant up-to-date, which translates into accountability and thus trust, control and even improved privacy (Prinsen, 2023).

Dynamic consent uses web-based technology features to overcome the problem of the lack of specific “real-time” information about individual research projects (Whitley et al., 2012). The platform must, however, be able to provide a flexible mechanism which provides different degrees of control to participants based on their personal preferences (Kaye et al., 2012). Dynamic consent endorses a process which emphasises continual re-contact with participants by providing real-time information on research projects, and allows for easy user-friendly revocation of any previously given consent (Steinbekk et al., 2013).

## 4 Dynamic consent and trust

Behavioural psychology has found that empowering participants establishes trust and approval, which results in greater participation (Dankar et al., 2020). Viewing participants as partners, as envisioned by dynamic consent, empowers participants and therefore improves trust. Furthermore, trust is fundamental in the successful use of data and dynamic consent may provide a flexible, transparent and user-friendly manner of providing information and maintaining trust (Williams et al., 2015).

Using consent as the basis of sharing data addresses the limitations associated with de-identification and anonymisation, while still respecting the autonomy of participants. Having a dynamic form of consent may allow participants to more readily provide or withdraw consent over time, while simultaneously providing information to the participants about how their material and data are used (Williams et al., 2015). Ongoing communication with researchers has also improved trust (Chen et al., 2020). Dynamic consent, a participant-centric consent approach, uses technology to allow ongoing engagement of research participants’ consent preferences as well as continual communication. Participants are also able to track and audit the use of their information, change their privacy settings and choose how and if they wish to be contacted. Dynamic consent thus enhances trust by giving the participants control over data flow (Williams et al., 2015).

Dynamic consent further fosters trust by providing mechanisms of accountability and transparency about the use of information and data as well as the sharing thereof (Prinsen, 2017). The improved scientific literacy offered by dynamic consent may also improve trust as it removes the fear of the unknown, so giving the participant confidence and, in turn, promoting better trust.

Dynamic consent also claims to allow for better adherence to regulatory systems (Kaye et al., 2015) and this sense of improved lawfulness may improve trust. Where participants feel that authorities bigger than themselves have approved an action, they may be more likely to believe that certain checks and balances are in place and that they and their goodwill are protected. Regulatory regimes may be considered as a vetting process, which allows the participant to better trust in the research process.

## 5 Dynamic consent, privacy and the protection of personal information

Trust, control and privacy are inextricably connected. If participants are able to have control over the protection of their privacy, they will be more likely to trust in the research endeavour and ultimately consent to participate in research. Attention must, however, be given to privacy and the benefit of using dynamic consent is highlighted.

### 5.1 Privacy

Privacy concerns may influence trust and, normally, participants wish to know who has access to their information, for what purpose, and who will make decisions in an ongoing manner since consent to participation is obtained prior to the start of participation. Participants need to be able to trust that their data is being used in accordance with their preferences (Horn et al., 2011).

Support for the sharing of data is often founded in privacy protecting safeguards, such as those envisioned by the Protection of Personal Information Act, which is discussed below. Where concerns have been raised in this regard, these were related to who the recipient of the information will be, anonymity and the type of information being shared, with indications of participants being more concerned the more personal the information is (Williams et al., 2015).

Current participant protection frameworks use de-identification of human biological materials and data, but according to Horn, Edwards and Terry (2011), participants are willing to make trade-offs for privacy if it means that they are able to stay connected to a study in which they are participating. De-identification severs the connection between the participant and the research and some participants may wish to stay connected by learning about the results of a study, for example,. The complete de-identification of material or data may also be harmful to participants as it hinders the return of incidental findings where appropriate, prevents researchers from obtaining follow-up information, and limits the participant’s ability to continually direct information (Horn et al., 2011). Dynamic consent may be indicated in these instances as it is premised on keeping participants connected and engaged to the research project and may thus offer a fair trade-off. Privacy enables the opportunity to negotiate how others access or use their information and the attitude towards these others is influenced by the level of trust in them (Schuler Scott et al., 2019). Furthermore, dynamic consent enables the return of findings and allows the researcher to obtain follow-up information.

Dynamic consent may also be useful in protecting participants from threats to their privacy as they are empowered to largely control access to their information. Four types of threats to privacy may be identified (Van der Geest et al., 2005): improper acquisition of information; improper use of information; improper storage and control of personal information; and privacy invasion. Dynamic consent and the use of participant preferences may be able to address all of these threats.

Privacy is often associated with ideas of self-identity, which are related to autonomy, and people have been shown to want control over their personal information and the decisions they may make about their data (Schuler Scott et al., 2019). Dynamic consent rests on participant engagement and facilitation of data, participation, and revocation of consent when needed. It protects tangible privacy interests rather than protecting privacy as an abstract concept (Schuler Scott et al., 2019). Claiming that dynamic consent is a privacy control means that it may be used by participants to manage how their information is shared and to what extent it may be shared even further (Schuler Scott et al., 2019).

### 5.2 Protection of personal information

The Protection of Personal Information Act of 2013 (POPIA) is the most recent addition to the collection of data protection Acts in South Africa and its purpose is to give effect to the constitutional right to privacy, to regulate how personal information may be processed, provide for rights and remedies to protect personal information, and to establish voluntary and compulsory measures to ensure respect, promotion, enforcement and fulfilment of the right to privacy (POPIA, s 2). It is suggested here that dynamic consent and the protection of personal information and privacy as provided for by POPIA are symbiotic and that dynamic consent may help overcome obstacles faced by health research because of the provisions of the Act.

POPIA requires specific consent for the processing of “personal information”, which is defined as (POPIA, s 

1): information relating to an identifiable, living, natural person, and where it is applicable, an identifiable, existing juristic person, including, but not limited to—

(a) information relating to the race, gender, sex, pregnancy, marital status, national, ethnic or social origin, colour, sexual orientation, age, physical or mental health, wellbeing, disability, religion, conscience, belief, culture, language and birth of the person;
(b) information relating to the education or the medical, financial, criminal or employment history of the person;
(c) any identifying number, symbol, e-mail address, physical address, telephone number, location information, online identifier or other particular assignment to the person;
(d) the biometric information of the person;
(e) the personal opinions, views or preferences of the person;
(f) correspondence sent by the person that is implicitly or explicitly of a private or confidential nature or further correspondence that would reveal the contents of the original correspondence;
(g) the views or opinions of another individual about the person; and
(h) the name of the person if it appears with other personal information relating to the person or if the disclosure of the name itself would reveal information about the person.

Some other definitions also need clarification in order to understand the discussion below. These definitions are (POPIA s 1):

1. Biometrics: a technique of personal identification that is based on physical, physiological or behavioural characterisation including blood typing, fingerprinting, DNA analysis, retinal scanning and voice recognition;
2. Consent: any voluntary, specific and informed expression of will in terms of which permission is given for the processing of personal information. It is suggested that dynamic consent meets all these requirements;
3. Data subject: the person to whom personal information relates. In the context of biomedical research, this is the participant;
4. Processing: any operation or activity or any set of operations, whether or not by automatic means, concerning personal information, including— (a) the collection, receipt, recording, organisation, collation, storage, updating or modification, retrieval, alteration, consultation or use; (b) dissemination by means of transmission, distribution or making available in any other form; or (c) merging, linking, as well as restriction, degradation, erasure or destruction of information;
5. Record: any recorded information— (a) regardless of form or medium, including any of the following: (i) writing on any material; (ii) information produced, recorded or stored by means of any tape-recorder, computer equipment, whether hardware or software or both, or other device, and any material subsequently derived from information so produced, recorded or stored; (iii) label, marking or other writing that identifies or describes anything of which it forms part, or to which it is attached by any means; (iv) book, map, plan, graph or drawing; (v) photograph, film, negative, tape or other device in which one or more visual images are embodied so as to be capable, with or without the aid of some other equipment, of being reproduced; (b) in the possession or under the control of a responsible party; (c) whether or not it was created by a responsible party; and (d) regardless of when it came into existence;
6. Responsible party: a public or private body or any other person which, alone or in conjunction with others, determines the purpose of and means for processing personal information. In the context of biomedical research, this is the researcher or research institution;
7. Special personal information: personal information as referred to in section 26, which includes health or biometric information of a data subject.

These definitions suggest that POPIA applies to numerous health research-related activities which range from the collection of health information, recording DNA analyses, storing health and biometric information and sharing such information (Thaldar and Townsend, 2021). Special personal information includes various types of research data such as genetic information and is subject to additional processing requirements. Considering the broad nature of biomedical research activities, it is suggested that it falls under the range of activities included under POPIA’s ambit. This application does not extend to the physical human biological material samples used in research but does include the information related to the sample, such as the participants’ particulars, and the data derived from the sample, such as genetic information which is then recorded (Thaldar and Townsend, 2021). Given that POPIA expressly aims to protect personal information, any information which has been de-identified beyond any chance of being re-identified does not fall within the scope of the Act.

In the Act, eight conditions which correspond to certain sections have been set for processing personal information. These conditions are:

1. Accountability: anyone who controls personal information of another person must appoint an Information Officer to ensure compliance with POPIA and its principles (POPIA, s 8);
2. Processing limitation: lawful processing, minimality of collected information, consent, justification and objection as well as collection of personal information directly from the data subject is provided for (POPIA, s 9–14);
3. Purpose specification: personal information must be collected for a specific purpose only and the person from whom the information is collected must be made aware of this purpose (POPIA, s 13 and 14);
4. Further processing limitation: this builds on the previous principle as it requires that where information must be further processed by a third party, processing must still be in accordance to the purpose specified (POPIA, s 15);
5. Information quality: the responsible party, the Information Officer, must take reasonable steps to guarantee that all the collected information is complete, accurate, not misleading and up-to-date. This must also be done in line with the purpose for which the information was collected (POPIA, s 16);
6. Openness: the Information Officer must be open regarding the collection of personal information. The Information Regulator as created by the Act must be notified if personal information is processed and where information is collected, the Information Officer must take reasonably possible steps to ensure that the data subject has been informed that their information will be collected (POPIA, s 17 and 18);
7. Security safeguards: the Information Officer must ensure that the integrity of the information over which they exert control is secured through technical and organisational measures (POPIA, s 19–22); and
8. Data subject participation: data subjects have the right to request that an Information Officer confirm whether they hold information on the data subject and they may further also request a description of this information (POPIA, s 23–25).

For this discussion, only conditions 2 to 4, 6 and 8 are of most pertinence. Conditions 1, 5 and 7 are also important but are administrative and technical and will be discussed briefly below as they relate to dynamic consent.

Condition 2, the processing limitation, requires legal grounds for the processing of information and the legal ground relevant to this discussion is consent by the data subject. As mentioned above, consent is defined as being voluntary, specific and informed in terms of the Act. The data subject must thus provide consent for the collection of information, recording of DNA analysis of a taken material sample, storing health or biometric information, using such information in conducting research, and sharing the information (Thaldar and Townsend, 2021).

Condition 3, the purpose specification, focuses on two types of processing: collection of information for a specific purpose, and retention and restriction of personal information records. For the collection of information, the purpose must be lawful, specific and explicitly defined. In the context of biomedical research and the very real possibility of secondary and future use of information and data, this is obviously problematic (Thaldar and Townsend, 2021). Information may then also not be retained for longer than needed to achieve this specific purpose. This may also be problematic but less so as an exception is provided by the Act in that information can be retained for a longer period with the condition that safeguards against the use of the records for any other purpose are provided for (POPIA, s 14).

Condition 4, the further processing limitation, requires that any further processing of information must be in line with the purpose for which the information was originally collected. Again, given the nature of biomedical research and the potential secondary and future use of data gathered during a research study, this condition is problematic. Consent may, however, be sought from the data subject to further process the information (Thaldar and Townsend, 2021).

Condition 6, openness, provides that a participant must be informed when their personal information is processed or collected.

Condition 8, participation, means that a research participant is entitled to request a researcher or research institution to provide them with the record or a description of the information held by them relating to the participant, which includes information on any third parties who have or have had access to their information. The participant may further request the correction or deletion of this information (Thaldar and Townsend, 2021).

Dynamic consent is in line with condition 2 as it is a form of consent which is “extra informed” as participants are informed about all new developments related to their material or data and it is also specific in that consent becomes fine-tuned and tailored by using participant preferences as was discussed above. Dynamic consent may be helpful in overcoming the requirements as set out by condition 3, as it allows the participant to specify the purpose for which their material or data may be collected. Dynamic consent also allows for preferences to be reset regarding secondary or future purposes for which their material or data may be used, thereby extending the period of time during which material or data may be retained. The same may be said of dynamic consent and condition 4. Dynamic consent is a platform of continual communication between the research participant and the researcher and this includes informing the participant of the use, or the collection or processing, of their material and data. This means that dynamic consent and condition 6 are symbiotic. Condition 8 is also enabled by dynamic consent as it is founded on the participation and engagement of the participant as a participant-centric initiative and by easier modification, withdrawal or revocation of consent.

In addition, dynamic consent may be useful in meeting the administrative and technical requirements set by conditions 1, 5 and 7. The requirement of an Information Officer who is responsible for seeing to POPIA compliance, thus protecting personal information, would be eased as the participant themselves will be involved in protecting their own information. Condition 5 which requires complete, accurate and up-to-date information would also be assisted by dynamic consent as the participant is enabled to change and update their information and preferences on an ongoing, real-time basis. Lastly, dynamic consent as an online, platform and interactive interface may help provide security safeguards.

#### 5.2.1 Exemptions from processing conditions

POPIA also allows for exemptions from the processing conditions and, again, dynamic consent may be able to ease some of the issues relating to this.

An exemption from any of the processing conditions may be granted where it may be shown that public interest in the processing of the information substantially outweighs any interference with the privacy of the participant. Section 37(2)(e) of POPIA includes research activities as a matter falling under public interest. This also includes health research (Thaldar and Townsend, 2021) which would in turn include biomedical research. However, showing that public interest substantially outweighs a constitutionally protected right, namely, privacy (Constitution of the Republic of South Africa, 1996, s 14) is more difficult (Thaldar and Townsend, 2021). This challenge may be overcome with the consent of the data subject but on the condition that specific consent was obtained from the data subject at the time of data collection. Although dynamic consent cannot be used retroactively to overcome this provision, if it were to be implemented now it would enable easier consent in future and would assist researchers with contacting participants to obtain consent. This would mean that using public interest as an exemption justification would be de-emphasised by the consent of the participant who then exerts control over their own privacy while exercising their constitutional right.

Special information may also not be processed unless consent has been obtained from the data subject or an exemption based on processing for historical, statistical or research purposes has been granted. This exemption will only be granted if the processing purpose serves a public interest, or if it is impossible or would involve a disproportionate effort to obtain consent for such processing and the necessary safeguards are in place to ensure that the data subject’s privacy is not disproportionately adversely affected. Again, although dynamic consent cannot change the past or do the impossible, if implemented now, it would be able to facilitate the obtaining of consent without causing disproportionate effort on the part of the researcher.

As illustrated, dynamic consent may be seen as able to symbiotically coexist with POPIA, assist in administrative and technical issues and even enable its functioning and application in a simplified manner in future. This means the protection of personal information as well as privacy benefit from the use of dynamic consent.

## 6 Limitations and implementation challenges

Although dynamic consent holds great promise, it is not without challenges or free of limitations. Implementing dynamic consent will require cultural changes both by participants and researchers and it will necessitate research relationships which are transparent, open and engaging, and which appreciate the role that participants play in research endeavours as the sources of material and information. Personal responsibility is problematic as this may place participants in a situation, real or perceived, that they are responsible for making decisions about complex issues that they do not fully grasp or are in no position to properly assess (Budin-Ljøsne et al., 2017). These systems will also have to accommodate participant responsiveness to the duration of a study in order to avoid withdrawal at a later stage in a study (Erlich et al., 2014). In addition, information fatigue will have to be guarded against (Teare et al., 2021).

A legitimate concern raised by dynamic consent relates to the creation of new ethical questions about co-responsibility and social exclusion. Representative uptake of participants may be an issue as groups of persons with lower socio-economic status may be less likely to engage in opt-in models of consent such as dynamic consent (Williams et al., 2015). Research Ethics Committees and Institutional Review Boards may not be familiar with dynamic consent which may hold up the approval of research projects and studies, thus negating the “quick reaction to change” advantage of dynamic consent (Budin-Ljøsne et al., 2017). Dynamic consent will also require the development of new policies and standards of practice. The consent mechanism and language will need to accommodate and adhere to existing regulatory schemes (Erlich et al., 2014).

This consent model requires technical capacities allowing research facilities and participants to engage and exchange information. For this reason, it will demand resources, including time, expertise, money and commitment from researchers, institutions and governments (Kaye et al., 2015). On an institutional level, implementation of dynamic consent may be difficult as it requires a certain e-infrastructure that is able to collect consent, to allow data preferences in order to direct the flow of such data, to capture a complete trail of data recipients, and to receive up-to-date lay summaries of research findings to return to the participants. Scalability is thus constrained by the provision and maintenance of such systems and infrastructures (Williams et al., 2015). Cost and maintenance of a dynamic consent platform may be very high as it requires staff with good communication and IT skills and may also require equipment where participants do not have their own devices (Budin-Ljøsne et al., 2017).

Unfortunately, heavy reliance on electronic communication strategies will exclude some individuals from participating in activities (Steinbekk et al., 2013). The implementation of dynamic consent also introduces issues which are not only of a technical nature but also concern the deeper ethics related to the digital divide (Wee et al., 2013). In developing countries such as many African nations, this may perhaps be the greatest impediment to implementation of a system of electronic dynamic consent. Access to technology is still largely exclusive and unequally distributed. Although numerous new methods of online engagement are becoming more commonplace, universal access is still a long way off. In addition to some participants not having access to the internet or devices, they may also not have the ability to use these technologies (Budin-Ljøsne et al., 2017) and so IT literacy may be problematic.

A further limitation also relates to research using samples and data already collected. It is, however, suggested that dynamic consent should not be seen as attempting to retroactively catch up with history—but should be implemented moving forward, for new projects starting off.

Some of these limitations may, perhaps, be overcome by using dynamic consent as complimentary to more traditional informed or broad consent processes, unique circumstances of a proposed research project permitting. Regardless, however, of the challenges in implementing a system of dynamic consent, it holds great potential for fostering and encouraging the rights and interests, trust and privacy of research participants.

## 7 Conclusion

This article discussed trust, dynamic consent and privacy in order to introduce and illustrate how dynamic consent may benefit and improve trust and privacy in biomedical research. Biomedical research is vital for increasing our understanding of health-related issues and holds great promise in this regard. However, to deliver on this promise, active participation of many human volunteers and distribution of data are needed. These participants have been protected by paternalism and by informed consent or by Institutional Review Board or Research Ethics Committee procedures.

However, the wide scope and nature of biomedical research challenges a one-size-fits-all approach to obtaining consent and to review mechanisms. Both informed consent and broad consent have been shown to be insufficient in biomedical research involving human participants. Researchers also need flexibility in conducting research in order to react quickly and therefore traditional approaches to the planning and conducting of biomedical research are unsatisfactory. Actively engaged research participants are becoming more commonplace in research and, accordingly, researchers have the potential to gain access to richer datasets and continued supporters of their research. When using trust-based frameworks, doing the right thing becomes easy and scientific progress becomes ever more possible.

While arguments have been made that trust is not necessary in biomedical research, studies over the past few decades noted that it plays an important role in the willingness of persons to participate in research and human participation in biomedical research is vital. Therefore, trust cannot be argued away.

This article also stated that contemporary data protection models rely mainly on de-identification but de-identification and standard data security measures are fallible. Trust and trust-enabling frameworks between participants and researchers may, however, be established by following principles whereby transparency creates trust, increased control enhances trust, and reciprocity maintains trust. Dynamic consent inherently entails all these principles.

Research participants are more likely to participate in research if they trust the researchers, the research organisation and the research project itself. The trust-consent relationship is one of reciprocity where consent is essential in nurturing trust–but it is the fruit of the tree, rather than its roots. Consent is also a mechanism which allows participants to exert control.

Since new research trends demand new models of consent, dynamic consent was introduced as such a new form of consent. Ethically, legally and theoretically, dynamic consent may benefit biomedical research and, by the participant empowering foundation of dynamic consent, trust may be established and fostered. Dynamic consent does this by:

• addressing the limitations associated with de-identification and anonymisation, while still respecting participant autonomy;
• enabling ongoing communication between participants and researchers;
• passing the control over data flow to participants;
• providing mechanisms of accountability and transparency for the use of information and data and the sharing thereof;
• improving scientific literacy; and even
• allowing for better adherence to regulatory systems.

Trust, control and privacy are inextricably connected and where participants are able to have control over the protection of their privacy they are likely to trust the research endeavour and, ultimately, participate in the research.

Current participant protection frameworks use de-identification of human biological materials and data, but it has been found that participants are willing to make trade-offs for privacy if they are then able to stay connected to a study in which they are participating. Dynamic consent was suggested in these instances as it is premised on keeping participants connected and engaged with the research project and this may therefore offer a fair trade-off. It also enables the return of findings and allows the researcher to obtain follow-up information.

This article illustrated how dynamic consent is in line with the information processing conditions provided for by POPIA and how it is symbiotic with the protection of personal information and may aid in the administration and technical requirements set by the Act.

Although dynamic consent cannot be used retroactively to overcome certain provisions of POPIA, it may enable easier consent in future and would assist researchers in contacting participants to obtain consent. It may also be of value in overcoming any disproportionate efforts in obtaining consent on the part of the researcher.

Although dynamic consent holds great promise, it is not free of limitations and these have been discussed. Regardless of these challenges, it still holds great potential for fostering and encouraging the rights and interests, trust and privacy of research participants. It should be strongly considered for biomedical research using human biological material and data.

## Data Availability

The original contributions presented in the study are included in the article/supplementary material, further inquiries can be directed to the corresponding author.
